# Divergent minute virus of canines strains identified in illegally imported puppies in Italy

**DOI:** 10.1007/s00705-020-04800-6

**Published:** 2020-10-08

**Authors:** M. Campalto, M. Carrino, L. Tassoni, G. Rizzo, M. C. Rossmann, M. Cocchi, P. De Benedictis, Maria Serena Beato

**Affiliations:** 1 Diagnostic Virology Laboratory, Istituto Zooprofilattico Sperimentale delle Venezie (IZSVe), Viale dell’Università 10, Legnaro, 35020 Padova, Italy; 2 Department for Infectious Diseases, International Animal Trade and Animal Health, Office of the Carinthian State Government, Carinthia, Austria; 3 Diagnostic Laboratory, Istituto Zooprofilattico Sperimentale delle Venezie (IZSVe), Via della Roggia, 100, Basaldella di C., 33030 Udine, UD Italy; 4 Laboratory for Viral Zoonosis, Emerging and Re-Emerging Pathogens, Istituto Zooprofilattico Sperimentale delle Venezie (IZSVe), Viale dell’Università 10, Legnaro, 35020 Padova, Italy

## Abstract

Minute virus of canines (MVC) belongs to the family *Parvoviridae*, genus *Bocaparvovirus*, and has been mainly described during enteritis episodes in young dogs. This study reports the characterization of four divergent MVC strains detected between 2012 and 2018, three of which were from dogs illegally imported into Italy, most probably from Eastern Europe, that cluster together phylogenetically but share low genetic similarity with the fourth MVC from an autochthonous dog and other available MVC sequences. Our data indicate that the introduction of genetically distinct MVC strains occurred through the illegal movement of dogs from a geographic area where a distinct MVC lineage was most likely circulating. Enforced surveillance of MVC in the dog population of Eastern Europe and its neighboring countries may shed light on, and eventually trace back to, illegal animal movements.

Minute virus of canines (MVC) belongs to the family *Parvoviridae*, genus *Bocaparvovirus*, and is antigenically distinct from canine parvovirus 2 (CPV2), which belongs to the genus *Protoparvovirus* and is a major causative agent of gastroenteritis [1]. Canine bocaparvoviruses (CBoVs) are non-enveloped single-stranded linear DNA viruses with a genome size of about 5.4 kb with three open reading frames (ORFs) coding for two non-structural proteins, NS1 and NP1, and two overlapping capsid proteins, VP1 and VP2 [1]. ORF1, located at the 5′ end, is 2,325 bp long and encodes NS1 (774 amino acids [aa]) [2], which is involved in replication, regulation of viral expression, and cytotoxicity [3]. ORF2, located at the 3′ end, is 2,112 bp long and encodes VP1 (703 aa) and VP2 (571 aa) [2]. The VP1 protein is critical for MVC infection, while VP2 mediates receptor recognition and nuclear translocation [3]. ORF3 (561 bp long) partially overlaps with ORF1 (191 bp) and ORF2 (17 bp) and encodes the NP1 protein (186 aa) [2], which plays an essential role in accumulating capsid mRNAs and proteins [3]. Genetic differences in the coding genes for NS1, NP1 and VP1/VP2 allow three genotypes to be distinguished: CBoV1, CBoV2, CBoV3. MVC corresponds to CBoV1 [1] and was first isolated in 1967 from the feces of a clinically healthy military dog [7]. MVC is distributed worldwide in domestic dogs of different ages. Its clinical significance and virulence are uncertain. It produces mild to unapparent infections, mainly enteritis, in puppies and is weakly pathogenic in adults [8]. Pneumonitis, myocarditis, lymphadenitis and hepatitis have been reported in dogs with MVC infection [9–11]. MVC may cross the placenta, causing early fetal death and birth defects [9, 10]. The undefined pathogenic role of MVC compared to CPV2 may account for the limited information available on MVC. In Italy, only one report is available describing a high-mortality episode in puppies of a shelter in Southern Italy in 2011 [9]. Novel cases of MVC infection have been detected in Italy at the Istituto Zooprofilattico Sperimentale delle Venezie (IZSVe) in dogs with signs of gastroenteritis, using a diagnostic approach based on the simultaneous detection of CPV2 and MVC. Such a diagnostic approach was implemented in the Austrian province of Carinthia during an interregional EU-funded project (BIO-CRIME, ITAT3002), allowing the responsible authorities to track down novel cases of MVC in dogs illegally imported into northeastern Italy. The present study reports the detection and genetic characterization of MVC strains identified in resident and illegally imported dogs in Italy between 2012 and 2018 whose genome sequences are divergent from all of the MVC sequences currently available in the GenBank database. Between 2012 and 2018, 540 samples (96 feces and 444 intestines) from dogs with diarrhea were submitted for parvovirus detection by private animal clinics to the IZSVe diagnostic laboratories, which also received carcasses for necropsy collected from northeastern Italy (Veneto and Friuli Venezia Giulia regions and the provinces of Trento and Bolzano). In the framework of the BIO-CRIME project, in 2018, 59 samples (20 intestines and 39 faeces) were collected. All samples were analyzed simultaneously for CPV2 and MVC by real-time PCR. A section of intestine (about 5 mm^3^) was homogenized in 0.8 ml of phosphate-buffered saline supplemented with antibiotics (PBS-A: 10,000 IU of penicillin G, 10 mg of streptomycin, 5000 IU of nystatin, and 0.25 mg of gentamicin sulfate per ml). Fecal samples were diluted 1:10 (w/v) in PBS-A, vortexed, and centrifuged at + 4 °C for 5 min at 14,000 × *g*. DNA was extracted from 200 µl of intestinal or fecal homogenate using a NucleoSpin Tissue kit (Macherey–Nagel, Germany). The following primers and probes targeting the VP2 gene of MVC and CPV2 were designed: MVC-for (5′-CTGCTCCTTTCTACATTCTC-3′), MVC-rev (5′-CATTATTGACCCACCCAC-3′), MVC-probe (FAM 5′-CATGAGGTGTTACGTACTGGGGAG-3′ TAMRA), CPV2-for (5′-GATCCAATTGGAGGTAAAACAGG-3′), CPV2-rev (5′-TTCTTTATCCCAAATTTGACC-3′), and CPV2-probe (FAM 5′-TGGTCCTTTAACTGCATTAAATAATGTACC-3′ TAMRA). Real-time PCR was carried out in a volume 25 µl containing 5 µl of DNA, 5 µl of Quantifast Pathogen Master Mix (5x) (QIAGEN, Germany), 1.5 µl of each primer (10 µM), 0.5 µl (10 µM) and 0.8 µl (10 µM) of probe for MVC and CPV2, respectively, 2.5 µl of Internal Control Assay (10x) (QIAGEN, Germany), and sterile ultrapure water to volume. The following thermal profile was used: Taq polymerase activation at 95 °C for 5 min followed by 40 cycles of denaturation at 95 °C for 15 s and annealing at 53 °C or 59 °C for 30 s for MVC and CPV2, respectively. All of the archived MVC-positive diagnostic samples were re-analysed by real-time PCR, and those with cycle threshold (Ct) values below 27 were sequenced by the Sanger method. Positive MVC samples identified during the diagnostic activity were subjected to virus isolation attempts. Five hundred µL of tissue homogenate was passed through a 0.45-µm filter and used to infect a confluent Madin-Darby canine kidney (MDCK, NBL-2, ATCC CLL-34) cell monolayer, maintained at 37 °C in a 5% CO_2_ atmosphere, and monitored daily. After three blind passages, MVC real-time PCR was carried out on cell supernatants to confirm the isolation of the virus. Tissues and cell cultures that were confirmed to be positive for MVC were used to determine the full MVC genome sequence. Six different endpoint PCRs were performed using primer pairs described by Shan et al. [11] covering the entire MVC genome with the exception of a small gap of 32 nucleotides (nt) in the NS1 gene (from nt 996 to 1,028). To cover this gap, an additional primer pair was designed ad hoc (MVC 550F, 5′-GGATGCCTGGTCCCGATAG-3′; MVC 1450R, 5′-ATAAGTTTGTTCCCGCCCGA-3′). The endpoint PCRs were conducted with 5 µl of PrimeSTAR GXL Buffer (5x) (Takara, Japan), 0.75 µl of each primer (10 µM), 2 µl of dNTPs (2.5 mM), 0.5 µl of PrimeSTAR GXL Taq polymerase (1.25 U/µl) (Takara, Japan), and 11 µl of sterile ultrapure water to 25 µl. The conditions for PCR were as follows: denaturation at 98 °C for 10 s, annealing at the primer annealing temperature for 15 s, and extension at 68 °C for 1 min, for 40 cycles. PCR products were subjected to electrophoresis in 7% acrylamide gels. Positive samples were subjected to Sanger sequencing using the same primers used for amplification. Complete sequences of CBoV1 available in the GenBank database were aligned, and a phylogenetic tree was constructed by the maximum-likelihood (ML) method, using the software IqTree-1.6.1, with the TMP3u + F + R2 nucleotide substitution model and of 100 replicates of bootstrap analysis. Further phylogenetic analysis was conducted for the individual ORFs in the MVC genome. For each analysis, the ORF sequences of the MVC strains identified in the present study (completely or partially sequenced) were aligned with those of the twelve complete genome sequences available in the GenBank database. Additional partial sequences were also included in the analysis ORF2. The nt substitution models for ORF1 (NS1), ORF2 (VP1/VP2), and ORF3 (NP1) were HKY + F + I, TVM + F + R5, and HKY + F + G4, respectively. The matrices of nt distances for the whole genome sequences and the VP1/VP2 sequences were calculated using MEGA software 6.06. Seven out of 540 diagnostic samples received at the IZSVe tested positive for MVC. Of the remaining 533 samples, 168 tested positive for CPV2 and negative for MVC, and 365 were negative for both. In detail, three out of the seven positive MVC samples were fecal samples, and the remaining four were intestinal samples. Two fecal samples collected in 2012 and 2017 and one intestinal sample collected in 2014 tested positive only for MVC; by contrast, one fecal sample collected in 2017 and three intestinal samples collected in 2014 (n = 2) and 2018 (n = 1) were positive for both MVC and CPV2. In 2018, seven out of the 59 samples analyzed in the framework of the BIO-CRIME project tested positive for MVC, CPV2, and coronavirus, and one sample tested positive for adenovirus type 1 (data not shown). Only three out of seven MVC-positive diagnostic samples identified and one sample out of seven from the BIO-CRIME project were subjected to virus isolation and Sanger sequencing, showing Ct values below 27. The sequenced diagnostic samples corresponded to two fecal samples collected in 2012 and 2017 (case numbers 2012-VIR-4033 and 2017-DIAPD-59116), one intestinal sample collected in 2018 (case number 2018-DIAPD-50120), which was positive for CPV2 as well, and one intestine from a puppy confiscated during the BIO-CRIME project (case number 2018-BIO-CRIME-4452) for a total of four MVC samples. Endpoint PCR generated amplicons of the expected size for four MVC-positive samples identified in 2012 (n = 1), 2017 (n = 1) and 2018 (n = 2), and four nearly complete sequences were reconstructed (Fig. 1a): MN947833_Minute_virus_of_canines_4033_Italy_2012 (Italy/4033/2012), MN947835_Minute_virus_of_canines_17DIAPD59116_Italy_2017 (Italy/59116/2017), MN947834_Minute_virus_of_canines_BIO-CRIME_4452_Italy_2018 (BIO-CRIME/2018), MN947832_Minute_virus_of_canines_18DIAPD50120_Italy_2018 (Italy/50120/2018). While Italy/4033/2012, Italy/59116/2017 and BIO-CRIME/2018 were identified in illegally imported puppies, Italy/50120/2018 was detected in an autochthonous six-year-old dog. Official authorities suspected that all illegal imports had originated in Eastern Europe, and at this time of writing, no other information was available. The genome organization of the four MVC isolates was similar to that of other CBoVs, containing three ORFs. In the present study, we were able to generate three nearly complete sequences of MVC genomes identified in three dogs with a suspected parvovirosis infection (Italy/4033/2012, Italy/59116/2017 and BIO-CRIME/2018), as well as a 2,637-bp fragment of a fourth MVC strain from a resident dog (Italy/50120/2018) (Fig. 1a). In detail: the complete ORF1, encoding the NS1 protein, of three of the four MVC isolates (Italy/4033/2012, BIO-CRIME/2018 and Italy/59116/2017, which had a gap of 17 nt) was sequenced and was 2325 bases in length, corresponding to 774 aa (Fig. 1a). The complete ORF2, 2112 bp long, encoding the VP1/VP2 proteins (703-aa VP1 and 571-aa VP2), was fully sequenced for two out of four MVC isolates: Italy/4033/2012 (with a small gap of 17 nt) and Italy/59116/2017. Partial ORF2 sequences were obtained for Italy/50120/2018 and BIO-CRIME/2018: missing 322 nt at 3′ end of VP2 and 26 nt at the 5′end and 326 nt at the 3′ end of VP2 (Fig. 1a). A full ORF3 sequence of 561 nt, encoding the NP1 protein (186 aa), was obtained for three out of the four MVC isolates: Italy/4033/2012, Italy/59116/2017 and BIO-CRIME/2018, but not for Italy/50120/2018 (Fig. 1a). The nearly complete genome sequences of Italy/4033/2012, Italy/59116/2017 and BIO-CRIME/2018, the two partial sequences of strain Italy/50120/2018, and the 12 whole MVC genome sequences available in the GenBank database were used for phylogenetic analysis. BIO-CRIME/2018 and Italy/59116/2017 were found to cluster together in a distinct monophyletic branch; by contrast, Italy/50120/2018 and Italy/4033/2012 clustered independently (Fig. 1b). To test the robustness of our analysis, we also generated phylogenetic trees using whole genome sequences trimmed to the shortest sequence length possible and including or excluding the region corresponding to the gap in the Italy/50120/2018 sequence. Although the topologies of the phylogenetic trees generated were slightly different, BIO-CRIME/2018 and Italy/59116/2017 strains always clustered together, and the corresponding nodes were supported by high bootstrap values (data not shown). The matrix of nt distances (Table 1) revealed a high similarity between BIO-CRIME/2018 and Italy/59116/2017 (99.21%) and showed less than 98% sequence identity to all of the full MVC sequences available in GenBank (Table 1). Conversely, Italy/50120/2018 and Italy/4033/2012 show high nt sequence similarity (> 98.51% identity) to MVC strains identified in Japan in 2008. In particular, the Italian MVC identified in autochthonous dogs (Italy/50120/2018) showed the most similarity to Japanese strains (AB518882, 99.01% identity; AB518883 and AB518884, 98.97% identity), while Italy/4033/2012 showed 98.51% nucleotide sequence identity to a Japanese strain identified in 2008 (AB518883) and to a Portuguese strain identified in 2011 (KY214446) (Table 1). The topologies of the phylogenetic trees based on each individual ORF (Fig. 2) was similar to that observed for the whole genome (Fig. 1b), with Italy/59116/2017 and BIO-CRIME/2018, both of which were obtained from smuggled puppies, clustering together in a separate new branch and the other two MVC strains falling in different branches (Fig. 2). The phylogenetic tree based on the ORF2 nt sequences encoding the VP1/VP2 proteins (Fig. 2c), was constructed using all 124 MVC sequences available in the GenBank database and included the only Italian MVC sequence available, Italy/285_11/2011 (JQ612703_Canine_minute_virus_strain_285_11_Italy_2011), for which only the partial VP1/VP2 sequence is present [9]. Interestingly, the phylogenetic analysis based on ORF2 sequences, highlighted that, among the MVC strains identified in the present study, the Italy/50120/2018 strain, from the autochthonous dog, is the only one that shares the highest nt sequence identity with Italy/285_11/2011 (99.07%) and is 100% identical to two Chinese strains identified in 2016 (MH051146 and MH051145) (Table 1). Moreover, the aa sequences encoded by the ORF1, ORF2 and ORF3 regions of the four MVCs identified were compared with those of the twelve complete MVC genome sequences available in the GenBank database. The aa sequence of the Italy/50120/2018 strain was analyzed for ORF1 and ORF2, but the ORF3 was not available. Several aa mutations were detected exclusively in the Italian MVC isolates, in particular in the NS1 and VP1/VP2 proteins, and are reported here for the first time. The VP1/VP2 of Italy/50120/2018 contained, a unique K253R mutation, and Italy/4033/2012 had D in position 468 instead of N, T, or G. In addition, Italy/50120/2018 had three unique aa mutations in the NS1 protein: Q275P, T395A, and E547V, and the BIO-CRIME/2018 strain had only two unique aa mutations in the NS1 protein: D380N and T in position 712 instead of A or S. The diagnostic algorithm for canine infectious diarrhea implemented at the IZSVe and by the partner laboratory in Carinthia allowed the identification of seven MVC infections out of 540 diagnostic cases of suspected parvovirosis (1.29%) between 2012 and 2018 that had otherwise gone undetected. This may indicate a low prevalence of MVC in northeastern Italy. Furthermore, this approach allowed the identification of three imported cases of MVC in the Italian territory. Similarly, seven out of 59 cases (11.86%) were detected in smuggled puppies investigated under the BIO-CRIME project in 2018. To date, only a few studies and fragmentary data are available on MVC. Its clinical relevance is considered low, although field evidence shows that MVC infection might be implicated in serious viral enteritis or even in hepatitis [11]. Genome sequencing of novel MVC strains is of considerable importance, since only a limited number of complete MVC genome sequences are publicly available. Therefore, the sequences generated in this study enrich the available information on the genetic characteristics of MVC strains, a necessary step to bridge the gap existing on the European continent. Moreover, this is the first report of the nearly complete ORF sequences of an MVC isolate identified in northeastern Italy that was probably introduced from Eastern Europe. Importantly, in the only Italian MVC case characterized so far (Italy/285_11/2011), only the sequence of VP1/VP2 was reported. Of note, two of the MVC sequences identified in this study (Italy/59116/2017 and BIO-CRIME/2018) appeared to be genetically unrelated to all of the other available sequences, forming a single distinct cluster, highlighting the uniqueness of these strains. The evidence that three MVC strains do not cluster with the Italian MVC isolates identified in autochthonous dogs in 2017 indicates a very likely introduction of novel MVC strains by illegal movement of dogs in 2012 and, more recently, in 2017 and 2018. The exact origin of such introductions cannot be established with confidence, but it is hypothesized that these viruses came from Eastern Europe, where a novel MVC lineage might be circulating. The genetic diversity of Italy/4033/2012 from the suspected illegally imported MVC strains identified in this study might indicate their different geographical origins. Interestingly, Italy/50120/2018, although detected in an autochthonous dog, exhibited a high degree of similarity to a Chinese strain, which may suggest that a MVC lineage was introduced into Italy from China and was then established in the Italian dog population. However, the absence of constant monitoring of MVC during gastroenteritis episodes limits any robust conclusion on the genetic characteristics of MVC circulating in Italy. Whether the MVC strains were directly responsible for gastroenteric signs or had the opportunity to be transmitted to autochthonous dogs deserves further investigation. Full-genome sequencing of identified MVC isolates jointly with enforced surveillance of MVC circulation in the Italian dog population may shed light on and eventually trace back illegal animal transport through the identification of newly introduced MVC lineages.

**Fig. 1:**
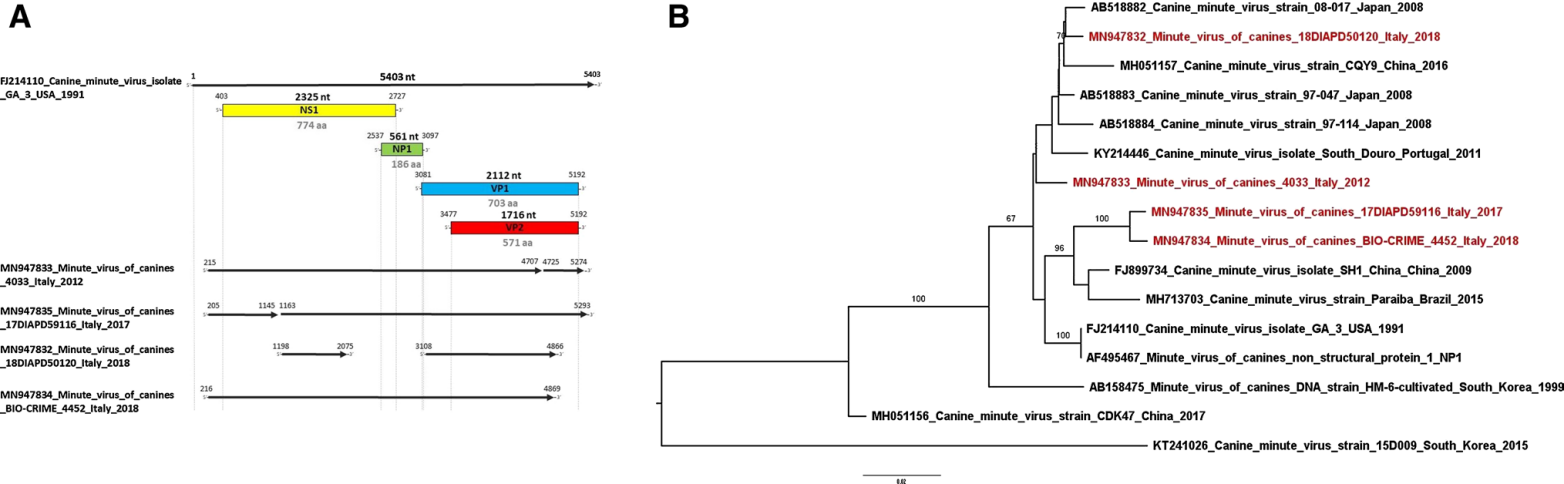
Genome organization of MVC and a phylogenetic tree based on nearly complete genome sequences. **(a)** Genome organization of MVC. Numbers flanking colored rectangles indicate the start and the end of the coding regions. Numbers above and below represent the size in nucleotides (nt) and amino acids (aa). Black arrows indicate the sequenced region of each strain characterized. **(b)** Phylogenetic tree based on the nearly complete genome sequences of strains Italy/4033/2012, Italy/59116/2017 and BIO-CRIME/2018, the two sequenced fragments of strain Italy/50120/2018 (red), and the twelve whole MVC genome sequences available in GenBank (black)

**Table 1:**
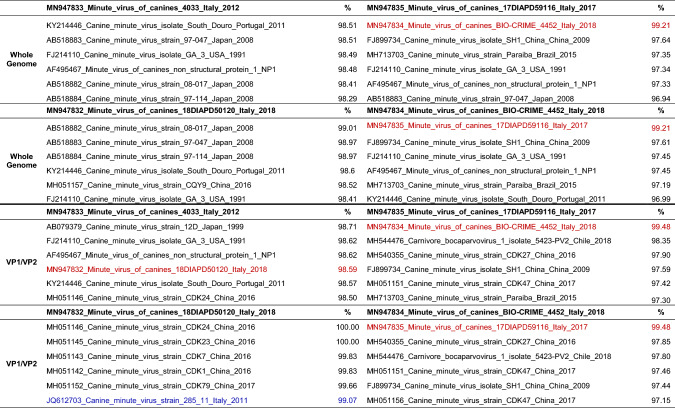
Whole-genome and VP1/VP2 nucleotide sequence similarity of MVC strains. Nucleotide sequence identity values (%) for the whole-genome and VP1/VP2 sequences of the MVC strains identified in the present study (red) in comparison to the most similar sequences available are shown. The Italian Italy/285_11/2011 strain is shown in blue

**Fig. 2:**
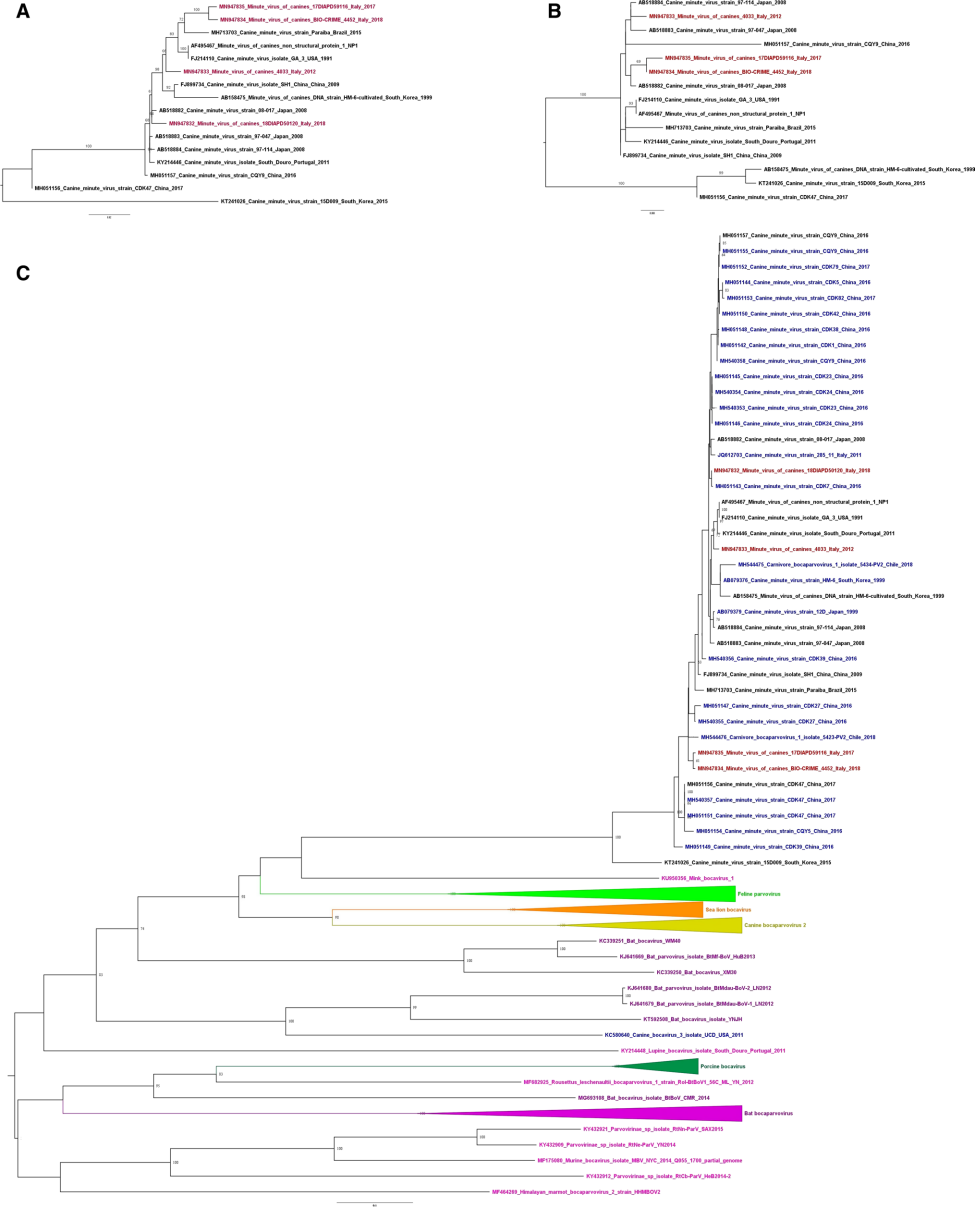
Phylogenetic trees based on individual ORFs of MVC strains. **(a)** Phylogenetic tree based on the NS1 (ORF1) region of the twelve genome sequences available in GenBank (black) and the four strains sequenced in this study (red). **(b)** Phylogenetic tree based on the NP1 (ORF3) region of the twelve genome sequences available in GenBank (black) and three of the strains sequenced in this study (red). **(c)** Phylogenetic tree based on the VP1/VP2 (ORF 2) region of CBoV1, CBoV2 and CBoV3. The sequences of the Italian strains characterized in the present study are shown in red, VP1/VP2 sequences derived from strains for which the whole genome sequence is available are shown in black, and the canine sequences for which only a VP1/VP2 sequence is available is shown in dark blue. The CBoV sequences from wild animals are in orange, light green and violet, and the porcine sequences are in dark green
